# Effects of Political Instability in Venezuela on Malaria Resurgence at Ecuador–Peru Border, 2018

**DOI:** 10.3201/eid2504.181355

**Published:** 2019-04

**Authors:** Robinson Jaramillo-Ochoa, Rachel Sippy, Daniel F. Farrell, Cinthya Cueva-Aponte, Efraín Beltrán-Ayala, Jose L. Gonzaga, Tania Ordoñez-León, Fernando A. Quintana, Sadie J. Ryan, Anna M. Stewart-Ibarra

**Affiliations:** Ministerio de Salud Pública del Ecuador, Machala, Ecuador (R. Jaramillo-Ochoa, J.L. Gonzaga, T. Ordoñez-León);; State University of New York Upstate Medical University, Syracuse, New York, USA (R. Sippy, D.F. Farrell, C. Cueva-Aponte, A.M. Stewart-Ibarra);; University of Florida, Gainesville, Florida, USA (R. Sippy, S.J. Ryan);; Universidad Técnica, Machala (E. Beltrán-Ayala);; Ministerio de Salud de Peru, Tumbes, Peru (F.A. Quintana)

**Keywords:** malaria, surveillance, migration, elimination, parasites, Venezuela, Peru, Ecuador, mosquitoes, political instability, vector-borne infections, Plasmodium spp

## Abstract

Mass migration from Venezuela has increased malaria resurgence risk across South America. During 2018, migrants from Venezuela constituted 96% of imported malaria cases along the Ecuador–Peru border. *Plasmodium vivax* predominated (96%). Autochthonous malaria cases emerged in areas previously malaria-free. Heightened malaria control and a response to this humanitarian crisis are imperative.

Malaria is a vectorborne parasitic infection caused by *Plasmodium* spp. and transmitted by *Anopheles* mosquitoes, characterized by fever and hemolysis with chronic and fatal potential (*1*). Despite substantial strides toward elimination in the Americas, malaria remains a major concern; ≈975,700 cases occurred and 138 million persons were at risk in 2017 (*2*). Most malaria cases in South America occur in the Amazon region, and *P. vivax* is more common than *P. falciparum* (*3*).

*P. vivax* and *P. falciparum* malaria were historically endemic to the Ecuador–Peru coastal border region. During 1990–2012, a total of 62,000 malaria cases were reported from El Oro Province, Ecuador, and 85,605 from Tumbes Region, Peru (*4*). Through vector control and active case surveillance and response, malaria was eliminated from El Oro Province in 2011 and Tumbes Region in 2012 (*4*). However, malaria cases elsewhere in Ecuador increased from 378 in 2013 (*5*) to 1,279 in 2017 (*6*). Peru and other countries in the region also reported increased malaria in 2017, indicating a major risk for reintroduction to elimination areas (*2*). In 2017, Venezuela alone accounted for more than half of all malaria cases in the Americas (*2*).

The public health sector in Venezuela is struggling with infectious disease epidemics, including malaria (*7*), despite a historically successful malaria control program (*3*). The worsening social and economic crisis has led to large-scale migration from and within Venezuela. The shortage of antimalarial drugs and lax in-country control efforts have exacerbated the situation, affecting countries throughout South America (*8*). Many people from Venezuela are migrating through Colombia and Ecuador to reach Peru and the southern cone of South America, stopping at various locations along the way (Figure). We report a series of imported malaria cases in migrants from Venezuela and the first autochthonous cases of malaria in the Ecuador–Peru border region since local elimination.

**Figure:**
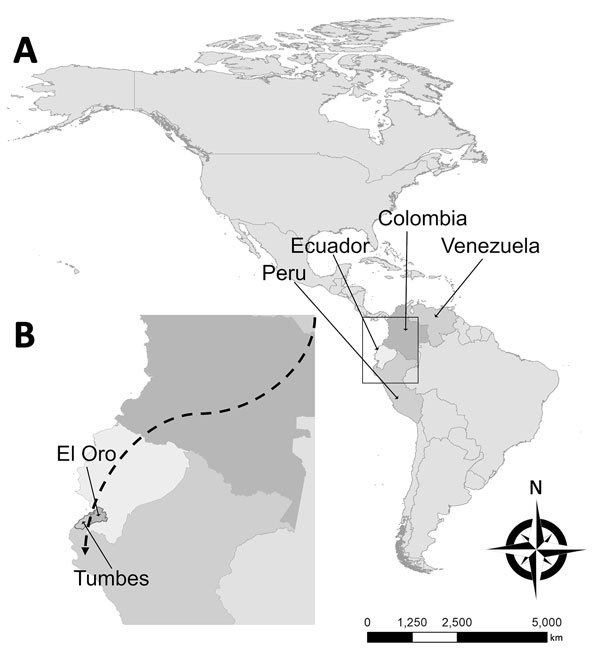
Probable migration route of imported malaria cases described in study of effects of political instability in Venezuela on malaria resurgence at the Ecuador–Peru border, 2018. A) Locations of the 4 countries along the migration route in South America; B) El Oro Province and Tumbes Region on the Ecuador–Peru border. The city of Huaquillas, Ecuador, is 70 km southwest of Machala, the location of the single autochthonous malaria case in this province. Huaquillas is the primary border crossing from Ecuador into Peru. Tumbes, the source of the 3 autochthonous cases in Peru, is the capital of Tumbes Region and is 22 km from the border. Dashed line in panel B broadly denotes the migration route taken from Venezuela through Colombia and Ecuador to Peru. Note the proximity of these countries and additional potential malarial resurgence through migration to Central America, the Caribbean, and the United States.

During February–November 2018, seven malaria cases (6 *P. vivax*, 1 *P. falciparum*) were detected in adults in El Oro Province and reported to the Ecuadorian Ministry of Health (Appendix). Five cases occurred in recent migrants from Venezuela, and 1 was imported from Peru. The most recent case (no. 7), reported in November 2018, was autochthonous. *Plasmodium* spp. infection was confirmed at the national reference laboratory in Guayaquil, Ecuador. Active surveillance within 1 km of each case-patient’s residence revealed no acute cases, and collateral thick blood smears were negative. Entomologic teams documented *Aedes aegypti* and *Culex* spp. mosquitoes in the homes but no *Anopheles* mosquitoes. The residences all had basic infrastructure and no history of malaria since local elimination in 2011.

During May–October 2018, a total of 20 *P. vivax* malaria cases were detected in adults in Tumbes Region and reported to the Peruvian Ministry of Health (Appendix). Seventeen cases occurred in Venezuelan migrants now living in the province, and 3 were autochthonous cases in persons residing in Tumbes. An epidemiologic investigation revealed that the autochthonous case-patients had no history of travel outside of Tumbes Region.

We cannot definitively state whether the migrants from Venezuela were exposed to malaria in Venezuela or during transit. Regardless, this population represents a highly vulnerable group with complex treatment issues. Malaria should be considered in the differential diagnosis for febrile patients from Venezuela and for local populations in nearby parts of South America. The transience of the migrant population presents treatment follow-up issues. The incubation period for *P. vivax* malaria is 12–18 days and, for *P. falciparum* malaria, 9–14 days. Case-patients (Appendix) often exhibited inadequately or untreated malaria. Imported cases are the likely source of the locally transmitted cases in Tumbes Region and El Oro Province because the primary mosquito vectors (*An. albimanus* and *An. punctimacula*) remain abundant in this area (*9*). Another concern is relapse of dormant *P. vivax* hypnozoites, which can occur up to several years after initial infection (*1*). Issues with primaquine (i.e., *CYP2D6-*poor metabolizers or hemolysis risk in patients with glucose-6-phosphate dehydrogenase deficiency) complicate treatment of dormant hypnozoites that cause relapse (*1*). A new treatment, tafenoquine, which still causes hemolysis in glucose-6-phosphate dehydrogenase deficiency, was recently approved in the United States as a single dose for prevention of *P. vivax* malaria relapse (*10*), although this medication might not reach at-risk groups in South America. Ecuador and Peru currently follow the Pan American Health Organization guidelines regarding primaquine use (https://www.paho.org/hq/dmdocuments/2011/TreatmentGuidelines-2nd-ed-2010-eng.pdf).

Local ministries of health responded quickly to these cases and implemented case surveillance. However, reductions in resources after elimination of local malaria transmission in 2011–2012 severely limited malaria control efforts in Ecuador and Peru. Imported cases of malaria at the Ecuador–Peru border region pose a serious threat of continued resurgence in local transmission. We urge international solutions for Venezuela’s humanitarian crisis and augmentation of infectious disease surveillance and control along migration routes and in surrounding regions.

Appendix Additional details on effects of political instability in Venezuela on malaria resurgence at the Ecuador–Peru Border, 2018.
