# Supplementary material for: Effects of Political Instability in Venezuela on Malaria Resurgence at Ecuador–Peru Border, 2018
Source: Emerg Infect Dis. 2019 Apr;25(4):834–6. doi: 10.3201/eid2504.181355 (PMC6433012; doi:10.3201/eid2504.181355)
Supplement: Appendix — Additional details on effects of political instability in Venezuela on malaria resurgence at the Ecuador–Peru Border, 2018. [file 18-1355-Techapp-s1.pdf]

Article DOI: https://doi.org/10.3201/eid2504.181355

Effects of Political Instability in Venezuela on Malaria Resurgence at Ecuador–Peru Border, 2018

Appendix

Clinical and Demographic Details for Malaria Cases from El Oro Province, Ecuador, and the Tumbes Region, Peru, in 2018

Case Descriptions: El Oro Province, Ecuador

Six cases of malaria were reported in El Oro Province from February to November 2018. Details are presented below and in Appendix Table 1.

Case 1: A 27-year-old woman from Venezuela, at 8 weeks' gestation, entered Ecuador in February 2018 with fever, chills, diaphoresis, and headache. She endorsed a prior history of incomplete treatment for *Plasmodium vivax* and *P. falciparum* on multiple occasions in Venezuela (Ciudad Bolívar). Thick blood smear revealed *P. vivax*. She received treatment with chloroquine, and subsequent thick blood smears (on days 7, 14, 21, 28) were negative. She continued with chloroquine preventive treatment until delivery.

Case 2: A 22-year-old man from Venezuela entered Ecuador in June 2018, with no prior history of malaria. His symptoms began 2 days after entry into Ecuador, and included fever, chills, diaphoresis, and headache. He was initially seen at an outpatient clinic and was prescribed amoxicillin and paracetamol. Six days later, he presented to a public hospital in the city of Machala, El Oro Province, Ecuador, with arthralgias, productive cough, and jaundice. While a cough is not usually thought of in malaria presentation, and in diagnosis, this has been reported in the literature (*1*). Diagnostic tests for dengue and leptospirosis were negative. Thick blood smear revealed *P. vivax*. He completed unspecified antimalarial treatment and follow-up thick blood smears (days 7, 14, 21, 28) were negative.

Case 3: A 41-year-old woman from Venezuela entered Ecuador in May 2018 with a past medical history of malaria on at least 16 occasions. She reported incomplete treatment due to lack of antimalarial medications in Venezuela and did not finish antimalarial treatment when *P. vivax* malaria was initially diagnosed in the city of Guayaquil, Ecuador (185 km north of Machala). She arrived in late July to the city of Machala, where *P. vivax* malaria was diagnosed again. She received treatment with chloroquine and primaquine for 7 days, and follow-up thick blood smears were negative. According to the Ecuadorean Ministry of Health team, she was treated an additional 14 days with primaquine to ensure killing of hypnozoites.

Case 4: A 51-year-old man entered Ecuador from the Peruvian Amazon (Canton Andoas) in August 2018 with fever, chills, and headache. He reported a history of malaria on 36 occasions. On August 22, *P. falciparum* malaria was diagnosed in Machala via thick blood smear. He was treated according to Pan American Health Organization guidelines (https://www.paho.org/hq/dmdocuments/2011/TreatmentGuidelines-2nd-ed-2010-eng.pdf).

Case 5: A 33-year-old man entered Ecuador in August 2018, having left Venezuela 10 days earlier. He had worked in the malaria-endemic Amazon region of Venezuela and had a history of malaria on at least 6 occasions. He reported treatment only with chloroquine in Venezuela. Symptoms (fever, chills, headache) began about 1 month after his arrival in Ecuador, and thick blood smear on September 17 showed *P. vivax*. He received unspecified antimalarial treatment. Follow-up thick blood smears (days 7,14,21, and 28) were negative.

Case 6: A 32-year-old man was diagnosed with *P. vivax* malaria in Venezuela (Bolivar state) 5 months prior, where he received treatment with chloroquine for 3 days and primaquine for 7 days. He left Venezuela in July 2018 and entered Ecuador 6 days later. His symptoms began in November of 2018, and included fever, chills, and headache. At that time, he presented twice to a health center in Machala, Ecuador, with unremarkable workup, including negative thick blood smears, and was treated symptomatically with paracetamol. One week later, he returned to the health center with jaundice, hepatosplenomegaly, and altered liver function test results. Thick blood smear

on November 14 showed *P. vivax*. He was treated with chloroquine and primaquine. Two of 4 follow-up thick blood smears (days 7, 14, 21, and 28) were negative. It was thought this was a relapse of *P. vivax* from liver hypnozoites; however, autochthonous transmission cannot be excluded.

Case 7: A 66-year-old man residing in the border city of Huaquillas in El Oro Province is thought to have the first autochthonous case of malaria in the province since 2011. The patient self-treated symptoms with paracetamol and sought medical care after 4 days with no improvement. A thick blood smear on November 28 revealed *P. vivax*, and the patient was treated with primaquine. Local control measures were implemented by the Ecuador Ministry of Health. No additional cases were found in the 1-km area around the patient's home.

Tumbes Region, Peru

Twenty cases of malaria were reported from May to November 2018 in the Tumbes Region, Peru (Appendix Table 2). Additional clinical details are unavailable at this time.

Reference

1. Anstey NM, Jacups SP, Cain T, Pearson T, Ziesing PJ, Fisher DA, et al. Pulmonary manifestations of uncomplicated *falciparum* and *vivax* malaria: cough, small airways obstruction, impaired gas transfer, and increased pulmonary phagocytic activity. J Infect Dis. 2002;185:1326–34. PubMed http://dx.doi.org/10.1086/339885

Appendix Table 1. Summary of malaria cases from El Oro Province, Ecuador, in 2018*

Case no.	Age, y/Sex	Origin	Pregnant	Month of entry to Ecuador, 2018	Symptoms	Month of diagnosis, 2018	History of malaria	Diagnosis	Follow-up
1	27/F	Venezuela	Yes	Feb	Fever, chills, diaphoresis, headache	February	Yes	*P. vivax*	Negative
2	22/M	Venezuela	NA	Jun	Fever, chills, diaphoresis, headache, arthralgias, productive cough, and jaundice	June	No	*P. vivax*	Negative
3	41/F	Venezuela	No	May	No details	July	Yes	*P. vivax*	Negative
4	51/M	Peru	NA	Aug	Fever, chills and headache	August	Yes	*P. falciparum*	Negative
5	33/M	Venezuela	NA	Aug	Fever, headache and chills	Septembe r	Yes	*P. vivax*	Negative
6	32/M	Venezuela	NA	Jul	Fever, headache and chills, jaundice, hepatosplenomegaly	November	Yes	*P. vivax*	Negative
7	66/M	Huaquillas	NA	Local resident	No details	November	Yes	*P. vivax*	Negative

*All cases were diagnosed by thick blood smear and received complete treatment. NA, not applicable.

Appendix Table 2. Summary of malaria cases from Tumbes Province, Peru, 2018*

Case no.	Age, y/sex	Origin	Symptoms	Date of diagnosis, 2018	Diagnosis
1	24/M	Venezuela	Fever, chills, diaphoresis, headache	May	*P. vivax*
2	59/M	Venezuela	Fever, chills, diaphoresis, headache	May	*P. vivax*
3	30/F	Venezuela	Fever, chills, diaphoresis, headache	Jun	*P. vivax*
4	53/F	Tumbes	Fever, chills, diaphoresis, headache	Jun	*P. vivax*
5	18/M	Venezuela	Fever, chills, diaphoresis, headache	Jul	*P. vivax*
6	20/F	Venezuela	Fever, chills, diaphoresis, headache	Jul	*P. vivax*
7	26/F	Venezuela	Fever, chills, diaphoresis, headache	Jul	*P. vivax*
8	22/M	Venezuela	Fever, chills, diaphoresis, headache	Jul	*P. vivax*
9	25/F	Venezuela	Fever, chills, diaphoresis, headache	Jul	*P. vivax*
10	35/M	Venezuela	Fever, chills, diaphoresis, headache	Jul	*P. vivax*
11	22/M	Venezuela	Fever, chills, diaphoresis, headache	Jul	*P. vivax*
12	48/F	Tumbes	Fever, chills, diaphoresis, headache	Jul	*P. vivax*
13	39/M	Venezuela	Fever, chills, diaphoresis, headache	Aug	*P. vivax*
14	21/M	Venezuela	Fever, chills, diaphoresis, headache	Aug	*P. vivax*
15	23/M	Venezuela	Fever, chills, diaphoresis, headache	Aug	*P. vivax*
16	18/M	Venezuela	Fever, chills, diaphoresis, headache	Aug	*P. vivax*
17	39/M	Venezuela	Fever, chills, diaphoresis, headache	Oct	*P. vivax*
18	22/M	Venezuela	Fever, chills, diaphoresis, headache	Nov	*P. vivax*
19	41/M	Venezuela	Fever, chills, diaphoresis, headache	Nov	*P. vivax*
20	35/F	Tumbes	Fever, chills, diaphoresis, headache	Nov	*P. vivax*

*Additional details unavailable.
